# Leveraging web search data in Germany to identify unmet needs of contraceptives on a population-based level: A longitudinal retrospective study

**DOI:** 10.1177/17455057241256919

**Published:** 2024-05-30

**Authors:** Charlotte Steiner, Hannah Wecker, Linda Tizek, Stefanie Ziehfreund, Sarah Preis, Kerstin Pfister, Viktoria Oberländer, Tilo Biedermann, Alexander Zink

**Affiliations:** 1 School of Medicine, Department of Dermatology and Allergy, Technical University of Munich, Munich, Germany; 2 Department of Gynecology and Obstetrics, University Hospital Ulm, Ulm, Germany; 3 Department of Obstetrics and Gynecology, Robert-Bosch Hospital, Stuttgart, Germany; 4 Division of Dermatology and Venereology, Department of Medicine Solna, Karolinska Institutet, Stockholm, Sweden

**Keywords:** contraception awareness, female contraception, infodemiology, non-hormonal contraceptive methods, pregnancy prevention, unmet needs

## Abstract

**Background:**

There are a variety of possible contraceptives available. While medical advice is an important resource for selecting the individual contraceptive, previous research has shown that the Internet has become an increasingly important source of health care information.

**Objectives:**

This study aims to identify key trends in contraception-related web searches in Germany and thus allows conclusions about preferences and unmet needs with regard to pregnancy prevention.

**Design:**

Longitudinal retrospective study.

**Methods:**

Google Ads Keyword Planner was used to identify contraception-related keywords and their search volume in Germany and all federal states between 2018 and 2021. The keywords were categorized based on gender, hormonal/non-hormonal, and different contraceptive methods. Search volume and categories were analyzed for temporal trends, regional differences, and underlying socioeconomic variables.

**Results:**

The 1481 contraception-related keywords corresponded to 15,081,760 searches. In total, a 56% increase in searches/100,000 inhabitants was observed. Highest mean search volume was observed in categories “woman,” “woman/non-hormonal” and “woman/non-hormonal/barrier,” respectively, and in the federal state Hamburg, while the lowest was seen in North Rhine-Westphalia.

**Conclusion:**

The increase in search volume reflects a high interest in contraception, particularly in non-hormonal female methods. This stands in contrast to the limited number of effective non-hormonal contraceptives available and points to an unmet need. In addition, the low search volume for male contraceptives demonstrates gender-specific responsibilities regarding family planning in German society.

## Plain language summary

Analyzing Google search volume to identify preferences and unmet needs in contraception in German society

Contraception is a topic that occupies many people in their daily lives. There is a wide range of different contraceptives available. The decision for a particular contraceptive can be made with the help of a consultation with a gynecologist or through other sources, such as the Internet. Research has shown that the Internet has become an important source for obtaining health information. This study aims to identify key trends in contraception-related web searches in Germany and thus allows conclusions about preferences and unmet needs with regard to pregnancy prevention. Google Ads Keyword Planner was used to identify contraception-related keywords and their search volume in Germany and all federal states between 2018 and 2021. The keywords were assigned to different categories based on whether the woman or the man uses the contraceptive, whether the contraceptive methods contain hormones or no hormones, and which exact contraceptive methods are associated with the search term. The search volume was analyzed in terms of trends over time, regional differences in Germany and socioeconomic variables. A 56% increase in searches per 100,000 inhabitants was observed. The highest mean search volume was observed in the category woman, followed by female non-hormonal contraceptives and within that category, the contraceptive method “barrier.” The study shows that there is a high interest in contraception, especially in non-hormonal female contraceptives. These results contrast with the limited number of non-hormonal and effective contraceptive methods available. In addition, the low search volume in the category man shows that women are the main responsible party in terms of contraception in German society.

## Introduction

Methods to prevent pregnancies play an important role in the daily lives of persons of childbearing age. Contraceptive methods can be divided into different categories. First, they can be classified into female or male application. However, most available contraceptives are designed for the female body. Second, different methods can be grouped into hormonal and non-hormonal methods. This means that the contraceptives contain either sex hormones or rely on a non-hormonal mechanism. The decision for or against the usage of a particular contraceptive method can depend on a wide variety of factors, for example, medical advice, efficiency, availability, or affordability.^1^ The history of contraception dates back many centuries and has gone through a substantial development. One of the first contraceptive methods was the usage of condoms made from intestines in the 16th century. Another significant achievement was the introduction of the first hormonal anti-baby pill in the 1960s.^2^ Since then, hormonal and non-hormonal contraceptives were developed with regard to their dosage and application to meet individual requirements and to reduce side effects, for example, the vaginal ring or wearable fertility tracking.^2–4^ Although the development of novel contraceptives is progressing, the methods of choice in Germany remain the anti-baby pill and the condom.^5^

Around 78% of Germans use the Internet as a healthcare information source. More than 80% of Germans used search engines regularly in 2018, with Google being the market leader with around 90% of all searches.^6,7^ It is, therefore, natural that the Internet is also increasingly used to gather information about contraception. While in 2001, only 1% of females and 10% of males aged 14–25 years used the Internet to inform themselves about sexuality and contraception, this number has risen to 56% and 60% in 2019, respectively.^8^

Despite significant advances in contraception, there appear to be unmet needs from users of contraceptive methods, for example, due to side effects, medical concerns, unreliability, or inconvenience.^9^ The aim of this study is to analyze contraception-related online search behavior in Germany to identify future trends, unmet needs, and developments in individuals’ interest for contraceptive methods.

## Material and methods

### Study design

**Figure 1.**
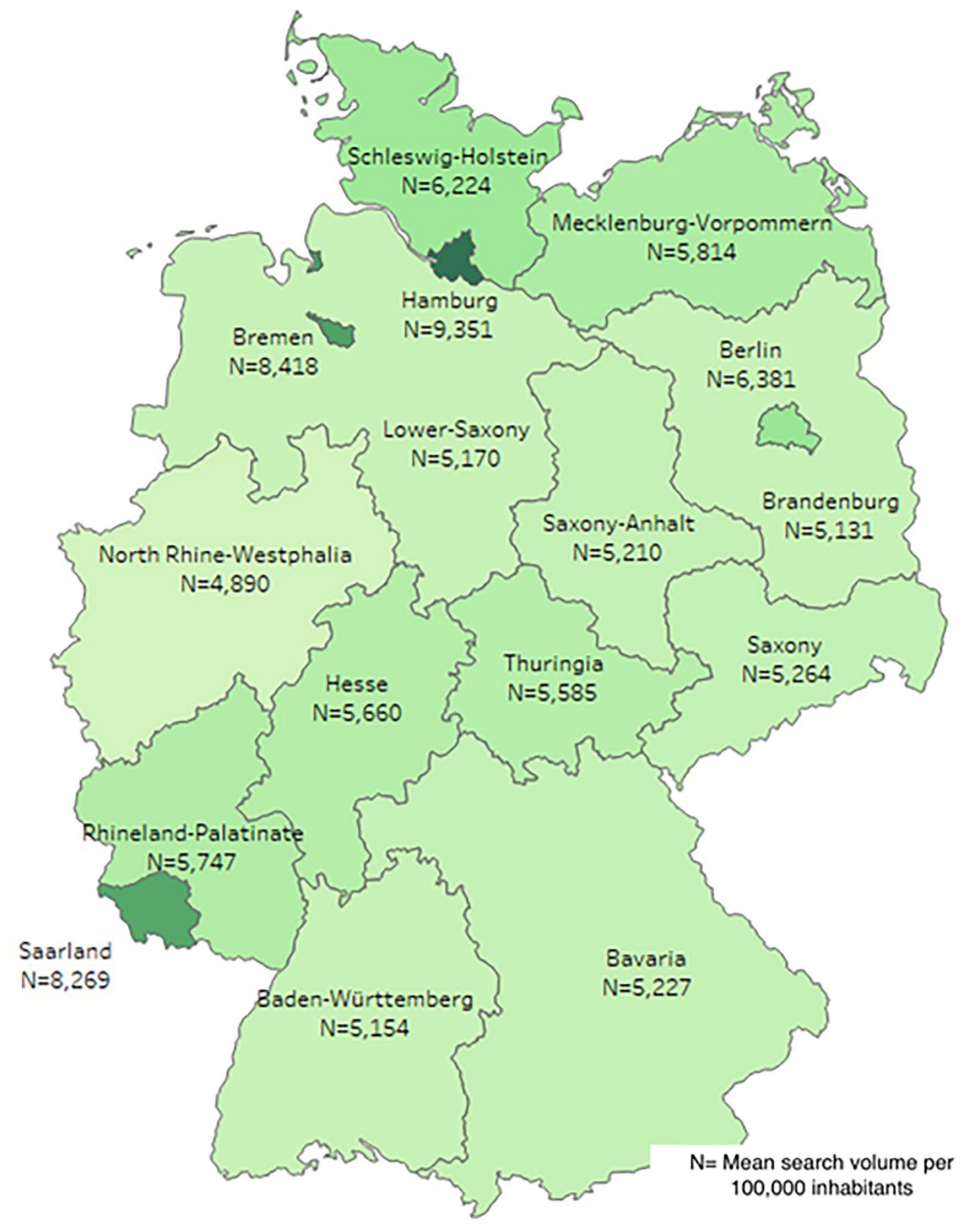
Mean Google search volume per 100,000 inhabitants for contraception in all federal states from January 2018 to December 2021.

Data for this longitudinal retrospective study were obtained from Google Ads Keyword Planner to analyze contraception-related search behavior for Germany as a whole and its 16 federal states between January 2018 and December 2021 (Figure 1). The German word for contraception (“Verhütung”) was chosen as the initial search term for this study. Google Ads Keyword Planner, a tool commonly used by online marketing campaigns, presents the total search volume (TSV) for any keyword related to the search term over a specific period. It is applicable for scientific inquiries as previously shown in numerous studies.^10–14^ The “Strengthening the Reporting of Observational Studies in Epidemiology (STROBE) statement” checklist was followed in this study. Moreover, only German language and German IP addresses were considered. As only publicly available data are used that cannot be attributed to any individual, there was no Institutional Review Board approval required.

Socioeconomic variables from 2019 (number of female citizens per 100,000 inhabitants, average population age, physicians per 100,000 inhabitants, and total fertility rate) were obtained from the Federal Institute for Research on Building, Urban Affairs, and Spatial Development.^15^

### Statistical analysis

Identified keywords were analyzed and split into “contraception” and “unavailable-alternatives” search terms. For example, “hormone-free contraception pill” references a pill that is not available on the market and therefore is not considered a valid contraceptive. All contraception-related search terms were classified and divided into three tiers of categories (Table 1)—first tier: (1) “gender neutral,” (2) “woman,” and (3) “man.” Within the categorization of Tier 1, all keywords (2) “woman” and (3) “man” included contraception-associated searches related to the respective gender, the category (1) “gender neutral” included gender non-specific terms and generalized search requests, for example “10 contraception methods.” Moreover, only search terms which clearly presented contraceptives which inhibit male reproductivity were assigned to the category “man” (e.g. pill for the man contraception or condom contraception), while other search terms were assigned to the category “woman” (e.g. the pill contraception). Within the three categories of the first tier, the search queries were assigned to the categories on the second tier: (1) “general” (e.g. alternative for the pill), (2) “hormonal” (e.g. the contraceptive ring), and (3) “non-hormonal” (e.g. all contraceptives without hormones). In the third tier, the search queries were assigned to different methodological approaches: (1) “general,” (2) “barrier,” (3) “chemical,” (4) “herbal,” (5) “invasive,” (6) “IUD”(intrauterine device), (7) “pill,” (8) “injection,” (9) “ring,” (10) “implant,” (11) “patch,” (12) “natural,” (13) “digital,” (14) “thermal,” and (15) “vasalgel.” All categories of tier three were described in detail in Supplementary Table 1. Keywords were matched to only one category within one tier.

**Table 1.** Overview of the three-tier categorization for contraception-related keywords for each category in Germany.

	Total 1481 contraception-related keywords; 15,081,760 searches, 4539 mean search volume per 100,000 inhabitants							
Tier 1 (No. keywords; MSV per 100,000 inhabitants)	Gender neutral (299 keywords; 883)		Woman (1062 keywords; 3494)			Man (120 keywords; 158)		
Tier 2 (No. keywords; MSV per 100,000 inhabitants)	General (271; 852)	Non-hormonal (28; 31)	General (266; 437)	Hormonal (202; 595)	Non-hormonal (594; 2462)	General (41; 113)	Hormonal (16; 7)	Non-hormonal (63; 39)
Tier 3 (No. keywords; MSV per 100,000 inhabitants)	NA	Barrier (8; 6)	General (222; 291)	General (28; 32)	General (106; 381)	NA	General (3; 1)	General (2; 2)
		Chemical (13; 23)	IUD (44; 145)	Pill (48; 48)	Digital (105; 161)		Pill (9; 3)	Herbal (5; 2)
		Invasive (3; 1)		Patch (24; 114)	Barrier (61; 755)		Injection (4; 2)	Barrier (20; 20)
		Herbal (4; 1)		Ring (45; 342)	Chemical (13; 6)			Chemical (1;0)
				Injection (24; 27)	Natural (155; 421)			Invasive (10; 2)
				IUD (9; 13)	IUD (147; 737)			Thermal (19; 10)
				Implant (24; 20)	Herbal (7; 2)			Vasalgel (6; 4)

Legend: IUD, intrauterine device; MSV, mean search volume; NA, not applicable; No., number.

Note: Figures for tier 3 might not add up exactly to tier 2 figures due to rounding errors.

To compare the search volume (SV) between different federal states and Germany, it was calculated per 100,000 inhabitants and reported with mean and standard deviation (SD).^16–19^ Spearman’s correlation coefficient (r) was calculated to determine possible correlations with mean search volume (MSV) per 100,000 inhabitants in 2019 and socioeconomic variables among all federal states. All statistical tests were performed two-sided and simulated with a 95% confidence interval (CI). To test for differences in monthly MSV among the federal states, a Kruskal–Wallis test was applied, followed by Dunn’s tests with Bonferroni correction as post hoc test, and the 95% CI was calculated using Monte Carlo simulation (n = 10,000). Statistical analysis was performed with IBM SPSS Statistics (Version 28, IBM Corporation, Armonk, NY, USA).

## Results

A total of 1488 keywords were investigated in this study. The 1481 contraception-related keywords correspond to 15,081,760 searches from January 2018 to December 2021 (Table 1). The most common search term was the German word for diaphragm (“Diaphragma”) with MSV of 633 per 100,000 inhabitants (SD = 167).

In Germany overall, search terms assigned to the category “woman” had the highest MSV per 100,000 inhabitants with 3494 (77%, SD = 664), while the MSV per 100,000 inhabitants for the category “man” was 158 (3%, SD = 63). Within the category “woman,” the category “non-hormonal” made up 70% (2462 searches/100,000 inhabitants, SD = 487) of the SV compared to the category “hormonal,” which made up only 17% (595 searches/100,000 inhabitants, SD = 118). The most searched method within non-hormonal female methods was “barrier” (31%, 755 searches/100,000 inhabitants, SD = 185), followed by “IUD” (30%, 737 searches/100,000 inhabitants, SD = 138) and “natural” (17%, 421 searches/100,000 inhabitants, SD = 61). The most searched methods within female hormonal application were “ring” with 57% and an MSV of 342 per 100,000 inhabitants (SD = 78), followed by “patch” (19%, 114 searches/100,000 inhabitants, SD = 27) and “pill” (8%, 48 searches/100,000 inhabitants, (SD = 7), and the least MSV was “IUD” (2%, 13 searches/100,000 inhabitants, SD = 1). The most searched male non-hormonal contraceptive method was “barrier” (50%, 20 searches/100,000 inhabitants, SD = 3), followed by “thermal” (27%, 10 searches/100,000 inhabitants, SD = 18) and “vasalgel” (10%, 4 searches/100,000 inhabitants, SD = 3). Male hormonal contraceptive methods made up 4% of the TSV (7 searches/100,000 inhabitants, SD = 1) (Table 1, Table 2).

**Table 2.** Most searched forms of contraceptives from 2018 to 2021 and obtained sociodemographic variables in 2019.

Federal states	Percentage of MSV per 100,000 inhabitants from 2018 to 2021				Sociodemographic variables in 2019			
	Most searched female form of contraceptives: non-hormonal	Most searched male non- hormonal contraceptive: barrier	Most searched female hormonal contraceptives: ring	Most searched female non-hormonal contraceptives: barrier/ IUD	Physicians per 100,000 inhabitants	Fertility rate	Average age (years)	No. women per 100,000 inhabitants
Baden-Württemberg	69	47	56	28, 30	138	1.57	43.2	50,304
Bavaria	70	48	60	28, 29	150	1.55	43.5	50,417
Berlin	69	44	44	29, 28	176	1.42	42.2	50,830
Brandenburg	63	50	42	26, 26	136	1.62	46.9	50,675
Bremen	65	56	34	24, 29	185	1.61	43.2	50,528
Hamburg	69	47	44	26, 30	180	1.47	41.7	51,064
Hesse	68	46	50	27, 30	140	1.54	43.5	50,617
Mecklenburg-Vorpommern	62	55	45	25, 26	153	1.58	46.9	50,712
Lower-Saxony	68	44	47	29, 30	140	1.6	44.3	50,616
North Rhine-Westphalia	69	46	54	30, 31	141	1.56	43.8	50,934
Rhineland-Palatinate	66	50	48	26, 31	138	1.56	44.5	50,585
Saarland	64	58	40	22, 31	157	1.46	46	50,914
Saxony	65	47	47	28, 25	150	1.59	46.5	50,719
Saxony-Anhalt	63	55	42	26, 26	146	1.60	47.6	50,799
Schleswig-Holstein	66	51	42	27, 29	148	1.54	45	50,999
Thuringia	63	54	43	26, 26	151	1.60	47	50,501

Legend: IUD, intrauterine device; MSV, mean search volume.

The seven “unavailable-alternative” keywords, representing less than 1% (MSV of 1 search per 100,000 inhabitants, SD = 1) of the TSV of 14,500 searches, were investigated separately. Three out of these seven keywords refer to hormonal contraceptives. However the words “hormone-free” were included in the same search query (e.g. hormone-free contraception pill). For these keywords, there was an increase in searches of 1.241% observed in 2018–2021.

**Figure 2.**
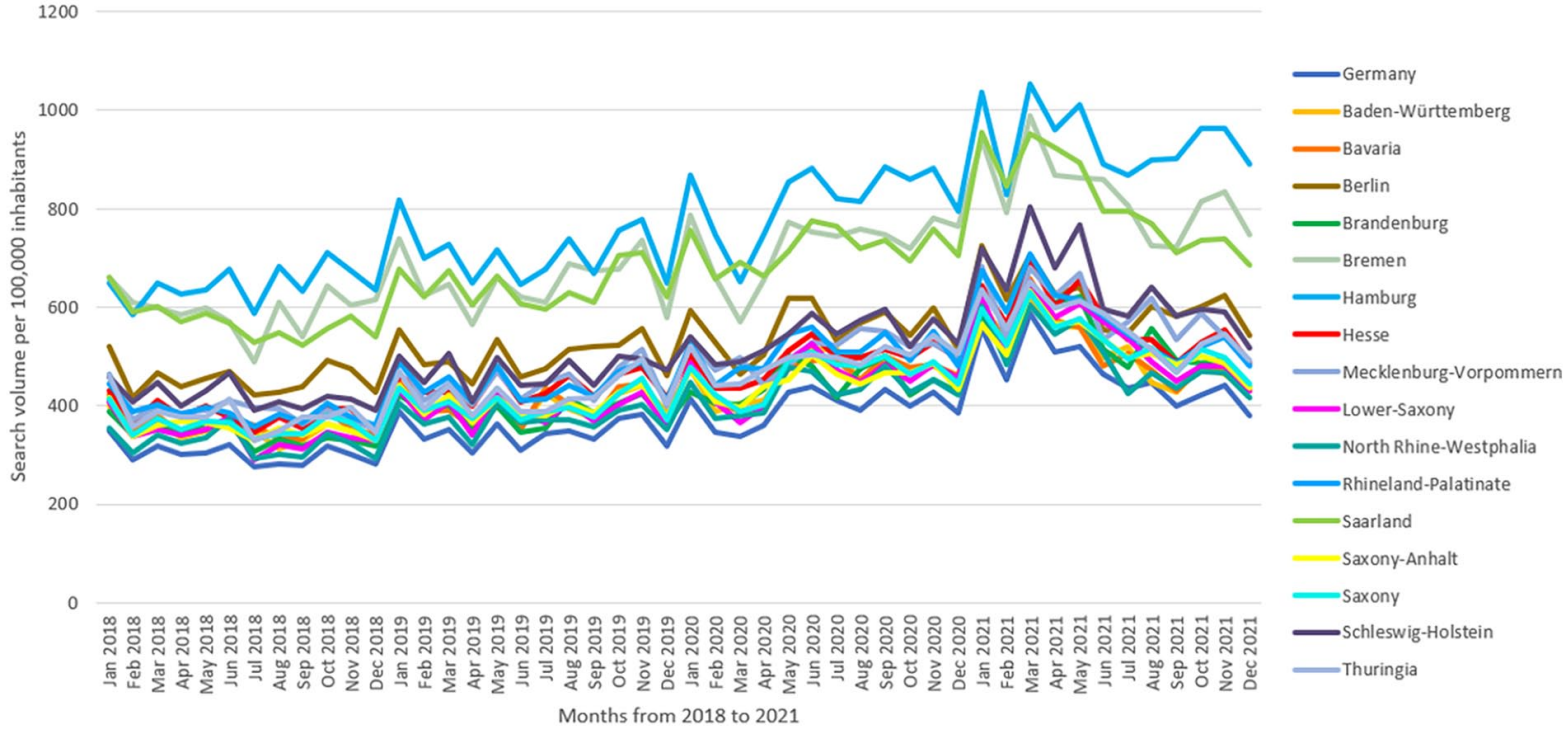
Time trend of the monthly contraception-related SV per 100,000 inhabitants in Germany from January 2018 to December 2021.

Differences in MSV per 100,000 inhabitants were found between the federal states (p < 0.001, CI: 0.000; 0.003). For example, Hamburg (n = 9336, SD = 1542), Bremen (n = 8402, SD = 1224), and Saarland (n = 8251, SD = 1265, Figure 1); the federal states with the highest MSV per 100,000 inhabitants, showed no significant differences to each other. However, all three showed significant differences to North Rhine-Westphalia, which had the lowest MSV per 100,000 inhabitants (n = 4884, SD = 911). The highest increase in searches of all federal states was observed in Brandenburg (56%), while the lowest increase in searches was observed in Berlin (35%) from 2018 to 2021. The compound annual growth rate was 16% in Brandenburg and 11% in Berlin (Figure 2).

### Time course of web searches

On average, 301 searches/100,000 inhabitants (SD = 22) conduced in 2018 increased to 469 in 2021 (SD = 63) which meant a 56% increase in searches nationwide, corresponding to a compound annual growth rate of 16% (Figure 2). The lowest SV per month was observed in July 2018 (276 searches/100,000 inhabitants), whereas March 2021 had the highest MSV (587 searches/100,000 inhabitants). Investigating the categories over time, the category “women” had the highest MSV per 100,000 inhabitants within all 4 years (77% in each year). The category “woman/non-hormonal/barrier” increased in SV per 100,000 inhabitants from 27% in 2018 to 31% in 2021, similar to “woman/hormonal/ring” (2018: 55%; 2021: 59%). Within all male non-hormonal contraceptive methods, “barrier” had a proportional SV per 100,000 inhabitants of 69% in 2018, reducing to 30% in 2021.

### Sociodemographic variables and statistical analysis

The MSV per 100,000 inhabitants and physicians per 100,000 inhabitants in 2019 correlated strongly (r = 0.758, p < 0.001, CI: 0.001; 0.406). The socioeconomic variable average population age (r = −0.333, p = 0.207, CI: −0.719; 0.210), female citizens per 100,000 inhabitants (r = 0.282, p = 0.289, CI: −0.263; 0.691), and total fertility rate (r = −0.499, p = 0.049, CI: −0.803; 0.011) revealed moderate-to-strong correlations with the MSV per 100,000 inhabitants in 2019.

## Discussion

The aim of this study was to analyze trends and unmet needs with respect to contraception. The conducted web search analysis shows an increasing interest in contraception from 2018 to 2021. The search focus was on female non-hormonal contraceptives, especially the diaphragm and the hormonal IUD, while only a small SV was assigned to male contraception.

The search behavior in Germany indicates unequal interest in male and female contraceptives. One interpretation of these results is that this is due to the limited number of options for males. Currently, there are only two methods of male contraceptives on the market—condoms and vasectomy. While condoms are not fully effective due to incorrect application, a vasectomy is an invasive procedure and is not reversible in every case.^20^ However, a promising approach is hormonal contraception for men, which represents a reversible option.^20^ Although most men agree that family planning should be a shared responsibility, the main responsibility for taking care of permanent contraception in heterosexual relationships falls on women.^21,22^ In general, there is a global need for innovative approaches for novel male contraceptives to achieve greater equity in family planning, which could also lead to a decrease in unintended pregnancies.^20^

The high proportion of searches assigned to non-hormonal female contraceptive methods showed that the interest lies in non-hormonal rather than hormonal contraceptives. This can also be seen in the disproportionate increase in “unavailable-alternatives” keywords (1.241%), including hormonal contraceptives with the appendix “hormone free.” However, according to a 2019 study by the German Federal Agency for Health Education in 2019, 47% of respondents named the hormonal pill as their contraceptive of choice, followed by the condom with 46%.^5^ This study shows that most women still use the pill, although the search behavior of Germans displayed that only 8% of searches within female hormonal contraception were assigned to the category “pill.” This could be driven by the fact that the pill is the oldest and best researched hormonal contraceptive.^23^ Moreover, oral contraceptives are only available on prescription, which could mean that the gynecologist is then the main information source. However, recently, the pill has become less popular. A study by the German health insurer “Techniker Krankenkasse” showed that in 2015, 73% of women aged 19 years chose the pill as the contraceptive of choice, while in 2020, the proportion decreased to 53%.^24^ A reason for the decline could be the potential side effects associated with the pill.^25,26^ Furthermore, there has been an increase in mistrust in hormonal contraceptives, for example, a concern for physical adverse reactions, altered mental health, and negative long-term effects, such as infertility.^27,28^ Both side effects and mistrust of hormonal contraceptives may also explain the high interest in non-hormonal alternatives. Contrary to the large share of Germans who choose the condom, this study shows a low and decreasing number of searches, reflected in the small proportion of TSV for the category “man/non-hormonal/barrier.”^29^ One can argue that condoms hold a similar role as the pill in society as a contraceptive that is well-known and widely used.

The high Internet interest of Germans in this female contraceptive method ring (57%) could indicate a preference for a lower hormone dosage indicated on the package. However, studies have shown that there is an equal or even higher risk for venous thrombotic events when applying vaginal rings due to different ways of metabolization.^30,31^ In comparison, the hormonal IUD showed little interest (3%) even though it releases lower daily hormone dosages than the pill.

The most searched non-hormonal method for women was intercourse with a barrier (31%) designed for female application, followed closely by non-hormonal IUD (30%). While the search behavior of Germans showed strong interest in these methods, the application in younger German society plays a minor role as shown in a study by the Robert Koch Institute.^29^ A reason for the low number of users of a barrier could be the limited effectiveness as 1–20 out of 100 women applying the method with contraceptive gel become pregnant.^32^ This underlines the fact that a consultation is important to apply the diaphragm correctly. However, the capacity for consultation is limited.^33^

The small number of non-hormonal and hormonal IUD users in German society could be explained by a lack of information. In general, women may lack information provided by gynecologists to make an informed decision about contraceptives.^34^

Hamburg showed the highest SV per 100,000 inhabitants, which could be explained by the youngest mean age (41.7 years of age) of all federal states and the highest proportion of women per 100,000 inhabitants (n = 51,064).^15^ A visit to the doctor can often result in a more in-depth search for information on the topics discussed, for example, via the Internet.^35^ This is supported by the positive correlation of a rising number of physicians per 100,000 inhabitants and the increasing SV.

Sometimes, women are dissatisfied with the decision-making process with their gynecologist and seek further information online.^36^ On social media platforms such as TikTok, discussions on hormonal and non-hormonal contraception are common. Pfender et al. found that TikTok content often focused on discontinuing hormonal contraception, neglecting discussions on alternative contraceptives. Positive aspects of hormonal contraception are mentioned less frequently compared to its negative aspects.^37^ Therefore, the information many users find via Google and social media must be placed in a critical context. This shows that in times of social media, it is even more important that healthcare journalists undergo good research training to provide readers with comprehensive, scientifically backed information, including quantitative insights into both positive and negative aspects.^38^

In general, the reasons for the abandonment of research and development in contraceptives are the already available effective and low-cost contraceptive methods and the difficulty to develop a novel method, which is as effective and affordable as already available contraceptives. The potential of novel methods is sizable due to the advancing development in chemistry, biology, medicine, and engineering, which is establishing a basis for further research and development for male and female contraceptives to fulfill unmet needs.^9^

### Limitations

This study holds limitations as it only analyzes the data from one search engine and only includes people with access to the Internet. However, Google is the market leader with around 90% of all searches, and around 92% of German households have Internet access.^6,39^ In addition, as all search queries are in German, the share of non-German language speakers was not considered. However, 80% of Germans speak only German in private households and therefore also search in German, while only 5% do not speak any German at home.^40^ However, future studies should consider non-German searches. Sociodemographic variables were limited by 2019. Demographic information on Google users is not provided by Google and the analyzed SV is estimated by the search engine company without verification by users. It is not possible to add one-to-one demographic data to the given SV, but correlating the sociodemographic data provided by the Federal Institute for Research on Building, Urban Affairs, and Spatial Development with the SV indicated the influence of demographics on search behavior. Moreover, it was only possible to determine possible correlations between physicians per 100,000 inhabitants and MSV per 100,000 inhabitants instead of the exact number of gynecologists per 100,000 inhabitants. This number was not provided by the Federal Institute for Research on Building. Within categorization, only search terms that clearly presented male contraception were assigned to the category “man.” It is assumed that this allows the best possible allocation of search terms to the gender-specific categories.

## Conclusion

The study demonstrates a growing interest in the overall topic of contraception. Contraceptive methods and society, more generally, are focused on the woman as the main applicant. Moreover, it is striking that within female contraceptives, non-hormonal contraceptive methods have by far the highest proportional SV, even though most women currently rely on hormonal contraceptives. Hence, there is a chance for intensified research on effective male and non-hormonal female contraceptives. Furthermore, the findings of this study can provide a prerequisite for future awareness, targeted research, and public health strategies going forward.

## Supplemental Material

sj-docx-1-whe-10.1177_17455057241256919 – Supplemental material for Leveraging web search data in Germany to identify unmet needs of contraceptives on a population-based level: A longitudinal retrospective study Supplemental material, sj-docx-1-whe-10.1177_17455057241256919 for Leveraging web search data in Germany to identify unmet needs of contraceptives on a population-based level: A longitudinal retrospective study by Charlotte Steiner, Hannah Wecker, Linda Tizek, Stefanie Ziehfreund, Sarah Preis, Kerstin Pfister, Viktoria Oberländer, Tilo Biedermann and Alexander Zink in Women’s Health

sj-docx-2-whe-10.1177_17455057241256919 – Supplemental material for Leveraging web search data in Germany to identify unmet needs of contraceptives on a population-based level: A longitudinal retrospective study Supplemental material, sj-docx-2-whe-10.1177_17455057241256919 for Leveraging web search data in Germany to identify unmet needs of contraceptives on a population-based level: A longitudinal retrospective study by Charlotte Steiner, Hannah Wecker, Linda Tizek, Stefanie Ziehfreund, Sarah Preis, Kerstin Pfister, Viktoria Oberländer, Tilo Biedermann and Alexander Zink in Women’s Health

## Declarations

**Ethics approval and consent to participate:** Ethical approval is not applicable as the study is based exclusively on publicly available data obtained through Google Ads Keyword planner. Consent to participate is not applicable as the study is based exclusively on publicly available data obtained through Google Ads Keyword planner.

**Consent for publication:** Not applicable

**Availability of data and materials:** The data that support the findings of this study are available from the corresponding author, A.Z., on reasonable request.
