# Supplementary material for: Leveraging web search data in Germany to identify unmet needs of contraceptives on a population-based level: A longitudinal retrospective study
Source: Womens Health (Lond). 2024 May 30;20:17455057241256919. doi: 10.1177/17455057241256919 (PMC11143866; doi:10.1177/17455057241256919)
Supplement: sj-docx-1-whe-10.1177_17455057241256919 – Supplemental material for Leveraging web search data in Germany to identify unmet needs of contraceptives on a population-based level: A longitudinal retrospective study [file sj-docx-1-whe-10.1177_17455057241256919.docx]

**Supplementary information**

**Supplementary Table 1: Explanation of categories within tier 3**

| **Categories within tier 3** | **Explanation** |
| --- | --- |
| Barrier | Prevents pregnancies by blocking the sperm and therefore inhibiting fertilization. These barriers can be applied to the female body, e.g., a diaphragm or to the male body, e.g., a condom |
| Chemical | Contains remedies which makes the sperm unable to fertilize |
| Invasive | Leads to infertility through an invasive procedure, e.g., vasectomy |
| Herbal | Prevents pregnancies with the aid of herbs, e.g., consumption of wild carrot seeds as a woman, which prevents implantation of the ovum |
| IUD | Contains hormones, copper or gold and anchors in the vault of the uterine wall |
| Pill | Contains hormones and requires regular oral intake |
| Patch | Releases a combination of hormones through the skin |
| Ring | Is inserted into the vagina and releases hormones |
| Injection | Contains progestins and is injected in a regular interval |
| Implant | Is placed under the skin and releases hormones |
| Digital | Includes all contraceptive methods which are applied with technical support, for example the assessment of the fertility status with the help of a wearable that is continuously worn on the skin and measures physiological parameters |
| Natural | Includes, for example, daily measurement of the body temperature or investigation of vaginal mucus production to determine the fertile window |
| Thermal | Includes all approaches of interrupting sperm production with the help of heat application |
| Vasalgel | Blocks the vas deferens (spermatic ducts) through an injection of a gel |
| General | Includes all other general search requests |
